# An Evolved Wavelet Library Based on Genetic Algorithm

**DOI:** 10.1155/2014/494319

**Published:** 2014-10-27

**Authors:** D. Vaithiyanathan, R. Seshasayanan, K. Kunaraj, J. Keerthiga

**Affiliations:** ^1^Department of Electronics and Communication Engineering, Anna University, Chennai 600025, India; ^2^Department of Electronics and Communication Engineering, Loyola-ICAM College of Engineering and Technology (LICET), Chennai 600034, India

## Abstract

As the size of the images being captured increases, there is a need for a robust algorithm for image compression which satiates the bandwidth limitation of the transmitted channels and preserves the image resolution without considerable loss in the image quality. Many conventional image compression algorithms use wavelet transform which can significantly reduce the number of bits needed to represent a pixel and the process of quantization and thresholding further increases the compression. In this paper the authors evolve two sets of wavelet filter coefficients using genetic algorithm (GA), one for the whole image portion except the edge areas and the other for the portions near the edges in the image (i.e., global and local filters). Images are initially separated into several groups based on their frequency content, edges, and textures and the wavelet filter coefficients are evolved separately for each group. As there is a possibility of the GA settling in local maximum, we introduce a new shuffling operator to prevent the GA from this effect. The GA used to evolve filter coefficients primarily focuses on maximizing the* peak signal to noise ratio* (PSNR). The evolved filter coefficients by the proposed method outperform the existing methods by a 0.31 dB improvement in the average PSNR and a 0.39 dB improvement in the maximum PSNR.

## 1. Introduction

Initially GA was developed to modify the coefficient sets of standard wavelet inverse transform which significantly improved the MSE for a given class of one-dimensional signals [1]. An investigation on evolutionary computation for image compression shows that it can be used to optimize wavelet coefficients and the transforms are independently trained and tested using three sets of images: digital photographs, fingerprints, and satellite images [2–4] and it was concluded that a better evolutionary progress towards an optimized reconstruction transform occurs when both the wavelet and scaling numbers are simultaneously evolved. Coevolutionary genetic algorithm based wavelet design for compressing fingerprint images was developed [5, 6] and the evolved wavelets outperform hand-design wavelet improving the quality of compressed images significantly. The suitability of the evolutionary strategy (ES) to implement it in Field Programmable Gate Array (FPGA) was investigated and the original algorithm was modified by cutting down several computing requirements [7–9]. The discrete wavelet transform (DWT) coefficients evolved using GA showed better compression and reconstruction of images with less MSE compared to 9/7 wavelet [10] and the detrimental effects of quantization for ultrasound images are compensated using the evolved transforms and its superior performance increases in proportion to the selected quantization level [11]. Moore et al. evolved matched filter pairs for deep space images that outperformed standard wavelets [12]. Even at three-level multiresolution analysis (MRA) transforms the evolved filters gives better compression performance for both photographic [3, 13] and satellite images [14, 15]. The adaptive embedded system developed by Salvador et al. performs an adaptive image compression in FPGA devices and finds the optimized set of wavelet filters in less than 2 minutes when the input image changes [16, 17]. Recently an adaptive fingerprint image compression (FIC) technique was carried out by evolving optimized lifting coefficients [18]. Evolving DWT filter coefficients separately for near-edge pixels and far-edge pixels have proven significant improvement in error when the images are reconstructed. Isolation of edge pixels can be done by the conventional edge detection algorithms like Sobel detector and a corresponding binary mask will separate the image into near-edge and far-edge objects [9].

### 1.1. Contribution

Primarily the input images are classified based on their frequency content, calculated by performing the DWT, and the corresponding method is detailed in Section 3.2. The training images are grouped according to the calculated average frequency metric and for each group separate DWT filter coefficients are evolved. The fitness function is formulated using PSNR value only but in the future we would like to extend the fitness function as a combination of PSNR,* energy compaction* (EC), and* structural similarity *(SSIM) index [19]. Perhaps, the authors believe that the optimization of wavelet filter coefficients with multiobjective fitness function formulated using PSNR, EC, and SSIM would yield a set of filter coefficients with better compression performance [20]. In this paper, the authors work is limited for the evolution of a library of wavelet filter coefficients for various groups of images considering the PSNR as the fitness function.

The rest of the paper is organized as follows.  Section 2 gives the sufficient background to understand the wavelets and genetic algorithm.  Section 3 pursues with the image classification based on frequency content. Detailed experimental setup for evolving DWT filter coefficients and the analysis of quality metrics of the reconstructed images are discussed in Section 4. The paper is concluded in Section 5 with the possible enhancements.

## 2. Background

The main objective of this paper is to evolve wavelet filter coefficients suitable for image compression for various groups of images classified according to their spatial frequency content. A detailed discussion about wavelets and genetic algorithm would be essential.

### 2.1. Wavelets and Image Compression

The wavelet is a multiresolution analysis tool widely used in signal and image processing. The analysis of the signal can be carried out at different frequencies and also with different time resolutions. It should be noted that there is a trade-off between frequency resolution and time resolution in wavelet. Hence the wavelet can be designed to provide good frequency resolution by giving off the time resolution and vice versa.

Discrete wavelet transforms (DWTs) are widely used for image compression as they have good compression capability. In particular, biorthogonal wavelets prove remarkable capabilities in still image compression. Perhaps the lifting scheme based DWT converts the high pass and low pass filtering operations into sequence of matrix multiplications and hence it proves to be efficient in terms of computation and memory.

#### 2.1.1. Discrete Wavelet Transform

**Figure 1:**
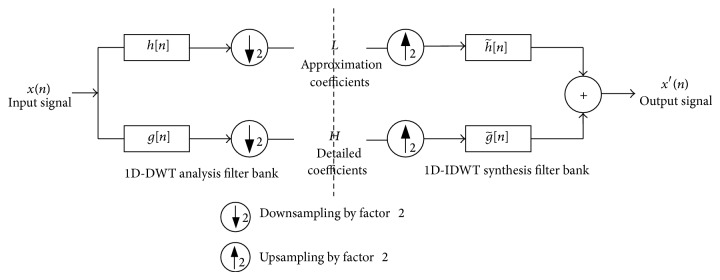
Single level wavelet transforms using convolution scheme.

The wavelet decomposition of the signal into different frequency bands is simply obtained by successive high pass and low pass filtering of the time domain signal. The original input signal *x*[*n*] is first passed through a half band high pass filter *g*[*n*] and a low pass filter *h*[*n*]. After the filtering process, half of the samples can be eliminated according to the Nyquist rule. The signal now has a highest frequency of *π*/2 radians instead of *π*. The signal *x*[*n*] can therefore be subsampled by 2, by discarding every other sample. This constitutes one level of wavelet decomposition as shown in Figure 1 and can mathematically be expressed as follows:

(1) $$\begin{array}{c} Y_{\text{High}}\left[k\right]=\sum_{n}x\left[n\right]\cdot g\left[2k-n\right]\\ Y_{\text{Low}}\left[k\right]=\sum_{n}x\left[n\right]\cdot h\left[2k-n\right]. \end{array}$$

The above procedure is followed in reverse order for the reconstruction. The signals are upsampled at every level and passed through the synthesis filters $\tilde{g}[n]$ (high pass) and $\tilde{h}[n]$ (low pass) and then added:

(2) x′[n]=∑k=−∞∞(YHigh[k]·g~[−n+2k])+(YLow[k]·h~[−n+2k]).

Fast wavelet transform (FWT) and/or Mallat's herringbone algorithm [21] which is a computationally efficient implementation of the DWT is used here to compute the wavelet coefficients.  Table 1 shows the CDF 9/7 filter coefficients for both forward and inverse DWT.

Wavelets are described by four sets of coefficients:

1. LOW is the set of wavelet numbers for the forward DWT,
2. HIGH is the set of scaling numbers for the DWT,
3. LOWR is the set of wavelet numbers for IDWT,
4. HIGHR is the set of scaling numbers for the IDWT.

#### 2.1.2. Lifting Based DWT and IDWT

Lifting scheme is a computationally efficient way of implementing DWT [22, 23]. The transform can proceed first with the* Lazy Wavelet*, then alternating* dual lifting* and* primal lifting* steps, and finally a scaling. The inverse transform proceeds first with a scaling, then alternating lifting and dual lifting steps, and finally the inverse lazy transform. The inverse transform can immediately be derived from the forward transform by running the scheme backwards and flipping the signs.

The polyphase decomposition of discrete low pass (LOW(*Z*)) and high pass (HIGH(*Z*)) filters are

(3) $$\begin{array}{c} \text{LOW}\left(Z\right)={\text{LOW}}_{e}\left(Z^{2}\right)+Z^{-1}{\text{LOW}}_{o}\left(Z^{2}\right)\\ \text{HIGH}\left(Z\right)={\text{HIGH}}_{e}\left(Z^{2}\right)+Z^{-1}{\text{HIGH}}_{o}\left(Z^{2}\right). \end{array}$$

The synthesis filters can be expressed through polyphase matrix:

(4) $$\begin{array}{c} T\left(Z\right)=\left(\begin{array}{cc} {\text{LOW}}_{e}\left(Z\right) & {\text{HIGH}}_{e}\left(Z\right)\\ {\text{LOW}}_{o}\left(Z\right) & {\text{HIGH}}_{o}\left(Z\right) \end{array}\right). \end{array}$$

And $\hat{T}\left(Z\right)$ can be analogously defined for the analysis filters.

Euclidean algorithm can be used to decompose *T*(*Z*) and $\hat{T}(Z)$ as

(5) $$\begin{array}{c} T\left(Z\right)=\overset{m}{\underset{i=0}{\prod }}\left[\begin{array}{cc} 1 & P_{i}\left(Z\right)\\ 0 & 1 \end{array}\right]\left[\begin{array}{cc} 1 & 0\\ U_{i}\left(Z\right) & 1 \end{array}\right]\left[\begin{array}{cc} K & 0\\ 0 & \frac{1}{K} \end{array}\right] \end{array}$$

(6) T^(Z−1)transpose=∏i=0m[10−Pi(Z−1)1][1−Ui(Z−1)01]×[1K00K].

**Figure 2:**
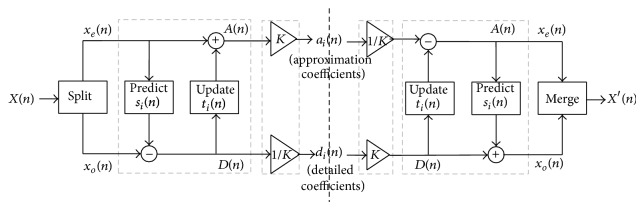
Discrete wavelet transforms using lifting scheme.

The discrete wavelet transform using lifting scheme consists of three steps as in Figure 2.

(1) *Split*: the original signal, *X*(*n*), is split into odd and even sequences (lazy wavelet transform)
  (7) $$\begin{array}{c} X_{e}\left(n\right)=X\left(2n\right) \end{array}$$
  (8) $$\begin{array}{c} X_{o}\left(n\right)=X\left(2n+1\right). \end{array}$$
(2) *Lifting*: it consists of one or more steps *m* of the form.
  (a) Predict/dual lifting: if *X*(*n*) possesses local correlation, then *X*_*e*_(*n*) and *X*_*o*_(*n*) also have local correlation; therefore, one subset (generally odd sequence) is used to predict the other subset (even sequence). Thus, the prediction step consists of applying a filter to the even samples and subtracting the result from the odd ones:
    (9) $$\begin{array}{c} D\left(n\right)=X_{o}\left(n\right)-P\left[X_{e}\left(n\right)\right], \end{array}$$
   where *P*[*X*_*e*_(*n*)] expresses that the value of *D*(*n*) is predicted by some combination of the value of *X*_*e*_(*n*).
  (b) Update/primal lifting: an update step does the opposite of applying a filter to the odd samples and adding the result from the even samples:
    (10) $$\begin{array}{c} A\left(n\right)=X_{e}\left(n\right)+U\left[D\left(n\right)\right]. \end{array}$$
   Eventually, after *m* pairs of prediction and update steps, the even samples become the low pass coefficients while the odd samples become the high pass coefficients.
(3) *Normalization/scaling*: after *m* lifting steps, scaling coefficients 1/*K* and *K* are applied to the odd and even samples, respectively, in order to obtain the high pass subband (*H*) and low pass subband (*L*).

Lifting scheme for biorthogonal 9/7 is as follows.

*Lifting Steps*

(11) $$\begin{array}{c} \text{Predict}\;P1:D_{o}\left(n\right)=X_{o}\left(n\right)+a\left[X_{e}\left(n\right)+X_{e}\left(n+1\right)\right]\\ \text{Update}\;U1:A_{o}\left(n\right)=X_{e}\left(n\right)+b\left[D_{o}\left(n-1\right)+D_{o}\left(n\right)\right]\\ \text{Predict}\;P2:D_{1}\left(n\right)=D_{o}\left(n\right)+c\left[A_{o}\left(n\right)+A_{o}\left(n+1\right)\right]\\ \text{Update}\;U2:A_{1}\left(n\right)=A_{o}\left(n\right)+d\left[D_{1}\left(n-1\right)+D_{1}\left(n\right)\right]. \end{array}$$

*Scaling*

(12) $$\begin{array}{c} X_{L}\left(n\right)=K\times A_{1}\left(n\right)\\ X_{H}\left(n\right)=\frac{1}{K}\times D_{1}\left(n\right). \end{array}$$

*a* = − 1.586134342, *b* = −0.052980118, *c* = 0.882911075, *d* = 0.443506852, and *k* = 1.2301741049/1.149604398.

**Figure 3:**
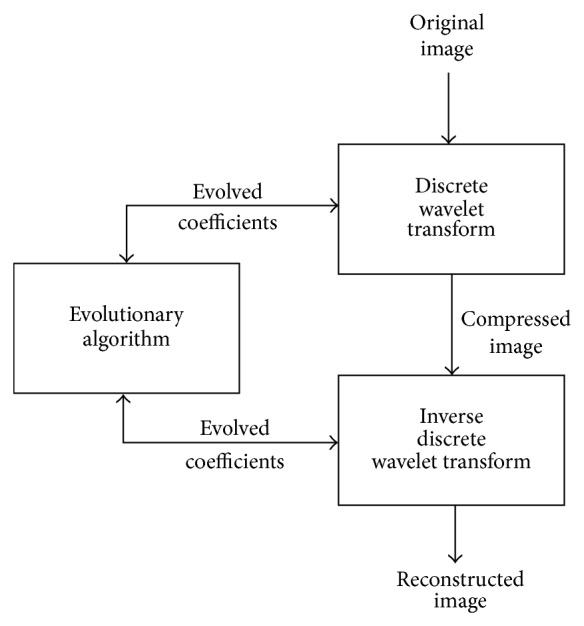
Evolutionary wavelet transform.

Thus by adapting wavelets to better suit the image, the performance of image compression can be increased. This adaptation is done by an evolutionary algorithm (EA) such as GA to improve the image reconstruction in the presence of quantization error by replacing the wavelet filter coefficients with a set of evolved filter coefficients. Evolutionary algorithm will evolve the best filter coefficients for the given image as shown in Figure 3.

### 2.2. Genetic Algorithm

**Figure 4:**
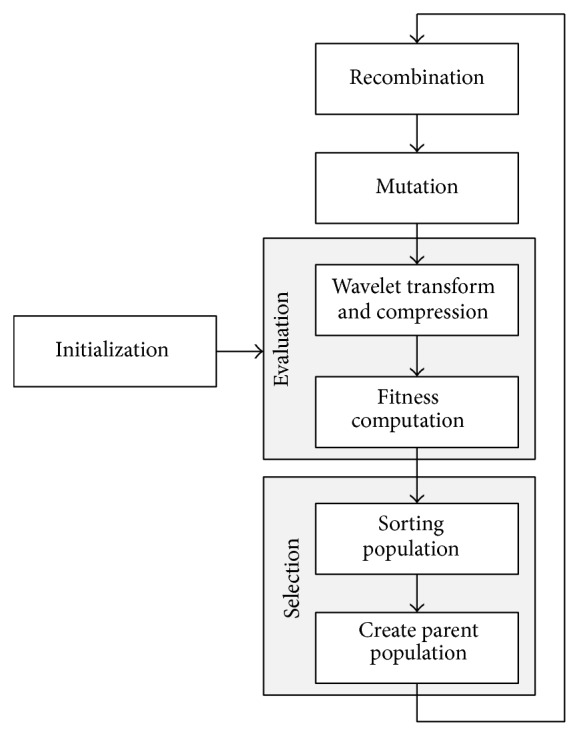
GA for evolving wavelet filter coefficients (Salvador et al., [7]).

Genetic algorithms (GAs) (first proposed by Holland) have frequently been used to solve a number of difficult optimization problems. GAs work by first creating a population of randomly generated chromosomes. Over a number of generations, new chromosomes are created by mutating and recombining chromosomes from the previous generation. Among the total population, the best chromosomes (solutions) are then selected for survival to the next generation based on some fitness criteria. The flow diagram of the GA for evolving wavelet filter coefficients is shown in Figure 4.

- Types of genetic algorithm.
  - Binary coded GA.
  - Real coded genetic algorithm (RCGA).

#### 2.2.1. Real Coded Genetic Algorithm

In RCGA the chromosomes are represented as real valued coefficients. The evolution of filters for image processing requires the simultaneous optimization of many real valued coefficients.

*Population Initialization*. The initial population includes one chromosome consisting of CDF9/7 filter coefficients. The remaining individuals are copies of the original wavelet filter coefficients multiplied by a small random factor. Additionally, 5% of the filter coefficients are negated. Each chromosome is composed of low pass filter coefficients, high pass filter coefficients, low pass filter reconstruction coefficients, and high pass filter reconstruction coefficients.

**Figure 5:**
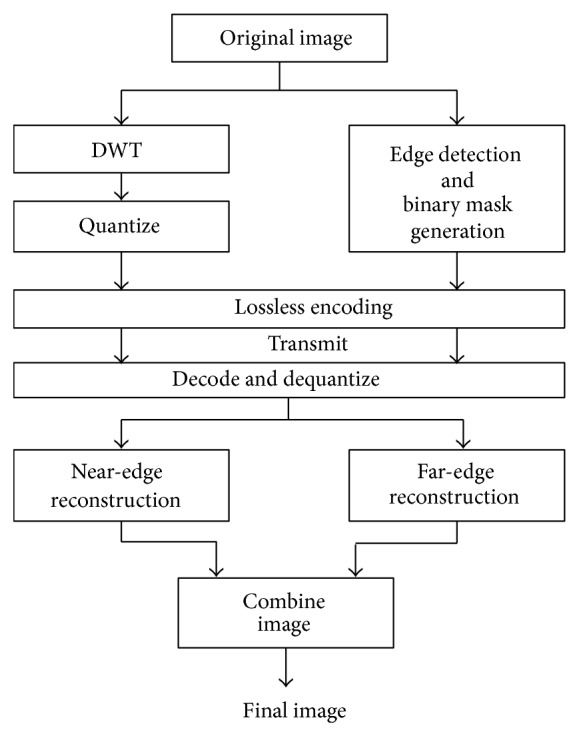
Image decomposition and reconstruction with evolved filters targeting edge-adjacent and non-edge-adjacent portions of image (Peterson and Lamont [9]).

*Evaluation*. The fitness of initial population is evaluated by first performing two-dimensional (2D) DWT on the test images and then the conventional decomposition and reconstruction (refer to Figure 5) is performed on the transformed coefficients and finally 2D IDWT is carried out to get the reconstructed image and the population is sorted according to the average fitness value.

Image quality (PSNR) and distortion (MSE) metrics are calculated between the original and the reconstructed image and the PSNR value is taken as the fitness measure. PSNR and MSE between the original (*X*) and reconstructed ($\hat{X}$) image of size *M* × *N* can be calculated using (13) and (14), respectively. Here *B* represents bits per pixel (bpp):

(13) $$\begin{array}{c} \text{PSNR}\left(\text{dB}\right)={20\log}_{10}\left(\frac{2^{B}-1}{\sqrt{\text{MSE}}}\right), \end{array}$$

(14) $$\begin{array}{c} \text{MSE}=\frac{{||X-\hat{X}||}^{2}}{MN}. \end{array}$$

An MSE = 0 in a reconstructed image indicates that $\hat{X}$ is a perfect reconstruction of *X*. Increasing values of MSE correspond to increasing error. 

*New Population Creation*. Once the population is evaluated for its performance, the new population is created from the parent population by the following.

- *Sorting* the population according to the evaluated fitness measure.
- *Selecting* the parents for reproduction by random/stochastic uniform selection methods.
- *Reproducing* the population for the next generation.

*Reproduction (Recombination and Mutation)*. The new population for next generation is created by crossover and mutation.

(i) * Elite*. It represents the number of best individuals which is copied from the parent population to the new population; Ne is elite count number.
(ii) * Heuristic Crossover*. The technique by which a child is created from two parents *P*_*i*_^1^ and *P*_*i*_^2^ biased in the direction of the parent with better fitness. Assuming *P*_*i*_^1^ has better fitness than *P*_*i*_^2^, then a child gene *C*_*i*_ is created as
  (15) $$\begin{array}{c} C_{i}=r\left(P_{i}^{1}-P_{i}^{2}\right)+P_{i}^{1}, \end{array}$$
 where *r* is randomly chosen in the interval [0, 1].
(iii) * Gaussian Mutation*. Mutation is required to avoid the premature population convergence in RCGA. Given a parent vector *P*, a new child vector *C* is created by *C* = *P* + *M*; *M* is based on Gaussian mutation, where the mutation shrinks in successive generations. Mutation Shrink rate controls the rate at which the average amount of mutation decreases. In early generations, the large variance permits quick exploration of the search space. Towards the end of the run, the variance is quite small, and the mutation operator makes very small refinements. If *k* is the current generation, “gens” is the total number of generations in the GA run. Thus the variance is calculated as
  (16) $$\begin{array}{c} {\text{var}}_{k}={\text{var}}_{k-1}\left(1-0.75*\left(\frac{k}{\text{gens}}\right)\right). \end{array}$$

*Proposed Shuffling Mechanism*. The probability of occurrences of the optimum solution increases by increasing the number of generation runs. The shuffling mechanism is primarily introduced to avoid the search algorithm getting struck at a local minimum [24]. Perhaps the search algorithm sometimes settles to the local minimum point and we call this phenomenon “positional effect” which can be avoided using the proposed “shuffling mechanism.” This shuffling operator totally changes the position of the elite chromosomes while getting replaced as new population for the next iteration. To a certain extent, this can make the search algorithm further visit some steepest points in the search space.

The proposed GA along with the genetic operators and shuffling mechanism is tested for convergence using few standard objective functions, namely, Rosenbrock function, De Jong's function, and Rastrigin's function and the results obtained show that the proposed GA is suitable for the optimization problem. The optimum solutions obtained by the proposed GA for the standard test functions are listed in Table 2.

#### 2.2.2. Genetic Algorithm Configuration for Evolving Global and Local Filters

The overriding goal of this research work is to develop a robust methodology for the evolutionary optimization of image transform filters capable of outperforming CDF 9/7 under conditions subject to quantization noise.

*Evolving Global and Local Filters*. Traditional image transformation algorithms are concerned with minimizing the global error between a reconstructed image and its original counterpart. Those transforms which are evolved to provide reconstruction over entire images tend to exhibit higher error rates near image object edges (Salvador et al., [8]).

*Improved Reconstruction through Edge Detection and Targeted Evolution*. Thus to improve the reconstruction of edges within an image, the image is reconstructed using two evolved image filters, globally evolved filters (Filters evolved using the entire image for fitness calculation to reduce errors in areas not adjacent to object edges.) and locally evolved filters (evolved using the edge-enclosing masks for fitness calculation to reduce error near object edges) and the two reconstructed images are combined by using binary mask which is generated by edge detection (canny edge detector) followed by thresholding. Figure 5 describes the process involved.

Initially the algorithm starts with separating the image into near-edge pixels and far-edge pixels using edge detector algorithm. Once the edges are detected, a binary mask is created which is a binary image which carries black pixels in the far-edge area and white pixels in the near-edge image area based on the threshold value. Hence, there are two classes of images (near and far edge) through which the evolutionary algorithm separately evolves suitable filter coefficients for the given set of training images. DWT is taken for both images using the respective filter coefficients and then it is quantized and encoded using lossless encoding algorithm like Huffman coding and transmitted. In the receiving side, the image can be reconstructed using appropriate wavelet filters and the individual near-edge and far-edge images are combined together to form a complete image.

### 2.3. Evolution of Wavelets

Evolution of wavelets can be carried out in the following ways.

- Convolution scheme
  (1) 9 variables (L1, L2, L3, L4, L5, H1, H2, H3, and H4);
  (2) 2 variables (LR3 and LR4).
- Lifting scheme
  (1) 5 variables (*a*, *b*, *c*, *d*, and *k*);
  (2) 1 variable (*k*).

#### 2.3.1. Convolution Scheme

*Population Initialization*. The initial population includes one chromosome consisting of CDF 9/7 filter coefficients. The remaining individuals are copies of the original wavelet coefficients multiplied by a small random factor. Additionally, 5% of the filter coefficients are negated. The initial configuration of the GA for each scheme is discussed in Table 3.

Each chromosome is composed of the following:

- low pass filter coefficients (9);
- high pass filter coefficients (7);
- low pass filter reconstruction coefficients (7);
- high pass filter reconstruction coefficients (9).

Initial population consists of the following:

- biorthogonal 9/7 (seed);
- *n* − 1 copies of 9/7 multiplied by a small noise factor.

*Evolving 9 Filter Coefficients*. In this method there are no constraints regarding the evolution of filter coefficients. The filter coefficients are allowed to evolve randomly and it is enough to evolve 9 filter coefficients L1, L2, L3, L4, L5, H1, H2, H3, and H4.

*Evolving 2 Filter Coefficients*. In this method, the wavelets are considered to be biorthogonal. Hence only LR3 and LR4 will be evolved here and all other coefficients will be derived as follows [25]:

1. $n=\sqrt{2}$;
2. LR2 = *n*/4 − LR4/2;
3. LR1 = *n*/4 − LR3;
4. L3 = (3*n*/4 − LR4)/(2 − 4*n*(LR4));
5. L4 = ((LR2^2^ × L3) + (LR3 × LR2) − (LR4 × LR1))/(2*n*/((LR3 × LR2) − (LR2 × LR1) − (LR4 × LR1)));
6. L2 = *n*/4 − L4;
7. L1 = −1 × ((LR1 × L2)/LR2);
8. L5 = 2 × (L4 − L3 + L2 − L1).

#### 2.3.2. Lifting Scheme

*Evolving 5 Variables*. In this method all the 5 parameters (*a*, *b*, *c*, *d*, *k*) are allowed to evolve randomly.

*Evolving 1 Variable*. In this method only *k* is allowed to evolve and all others are derived as follows [26]:

1. *a* = (1 − 2*k*)/4(*k* − 1);
2. *b* = −(*k*−1)^2^;
3. *c* = 1/4*k*(*k* − 1);
4. *d* = *k* ^3^ − (7/4)*k* ^2^ + *k*.

## 3. Image Classification Based on Frequency Content

**Figure 6:**
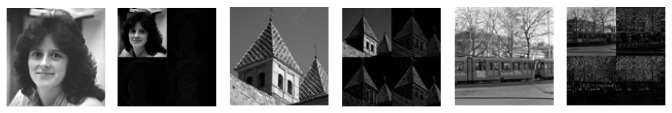
Relationship between detail subbands and image content.

The DWT filter coefficients evolved for images with smooth regions might not suit well for edge and texture rich images. Also, it is not practical to construct the optimal wavelet for each image as an online process in spite of the best compression with the evolved filter coefficients. Hence all the test images are classified according to the complexity of the images (edges and textures) and optimal wavelets are evolved for each class to build a wavelet library offline. The quality of the DWT-based compression method for remote sensing images is effectively assessed using a gradient based approach by classifying image pixels according to the gradient magnitude and texture complexity thus proving the importance of the edges and textures in an image [27]. Hence we propose a systematic approach to find the edges and textures of the image by using the DWT itself. The high frequency subbands of transformed image will depict the edge and texture content in an image. Texture rich images will have more coefficients in the high frequency subbands as depicted in Figure 6 (3 cases are considered). This implies that the images can be classified by looking into the high frequency subbands. Thus the* Frequency_Mean* (summation of absolute averages of all the high frequency subbands) in frequency domain of an image is taken as measure to classify the images.

### 3.1. Test Images

**Figure 7:**
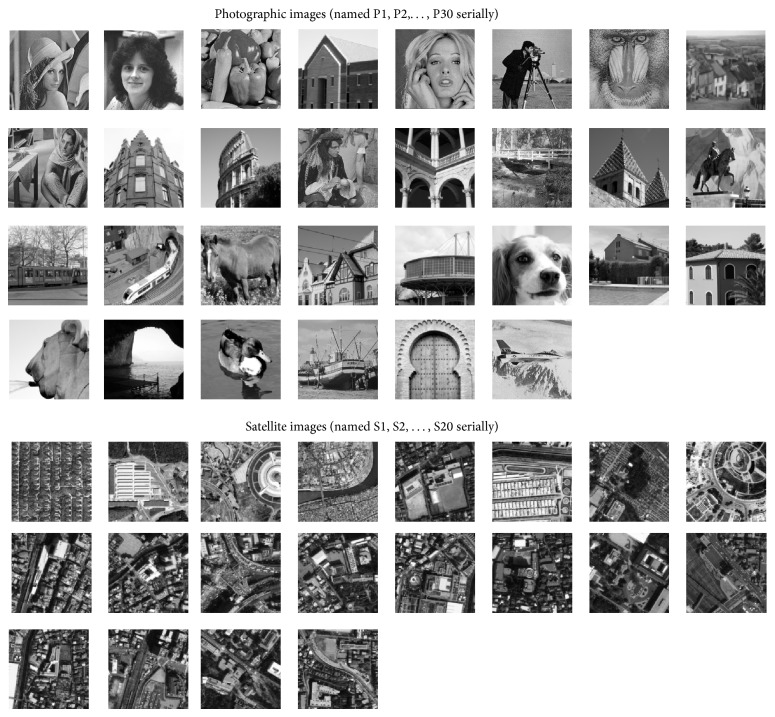
Test images.

We have taken 50 images as shown in Figure 7 for each run and those 50 images are classified into six groups (G1, G2,…,G6) and the wavelets are evolved separately for each group and all of them are classified according to* Frequency_Mean* (*F_*MEAN).

### 3.2. Calculation of* F*_MEAN

**Figure 8:**
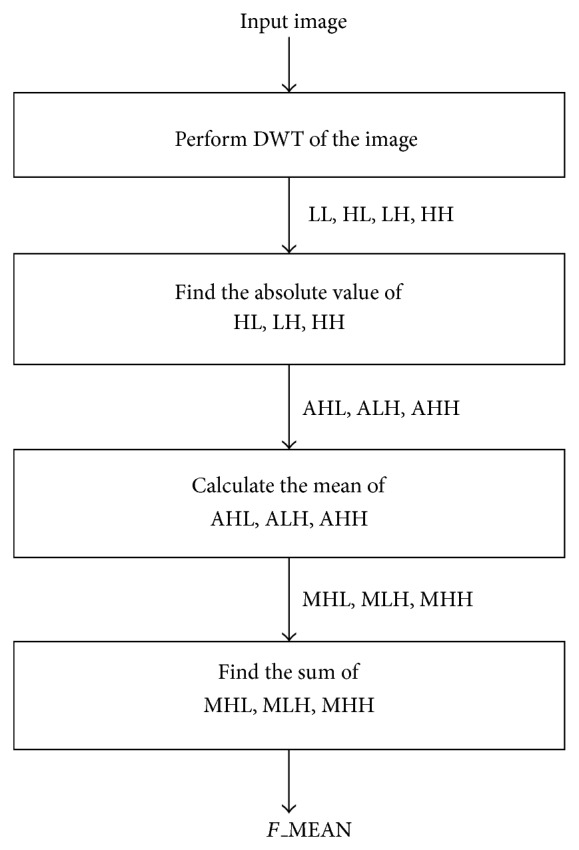
Calculation of* F*_MEAN.

The* F_*MEAN is calculated using the steps followed in the Figure 8 and the calculated* F_*MEAN are shown in Table 4 for the considered 50 test images.

### 3.3. Classification of Images

**Figure 9:**
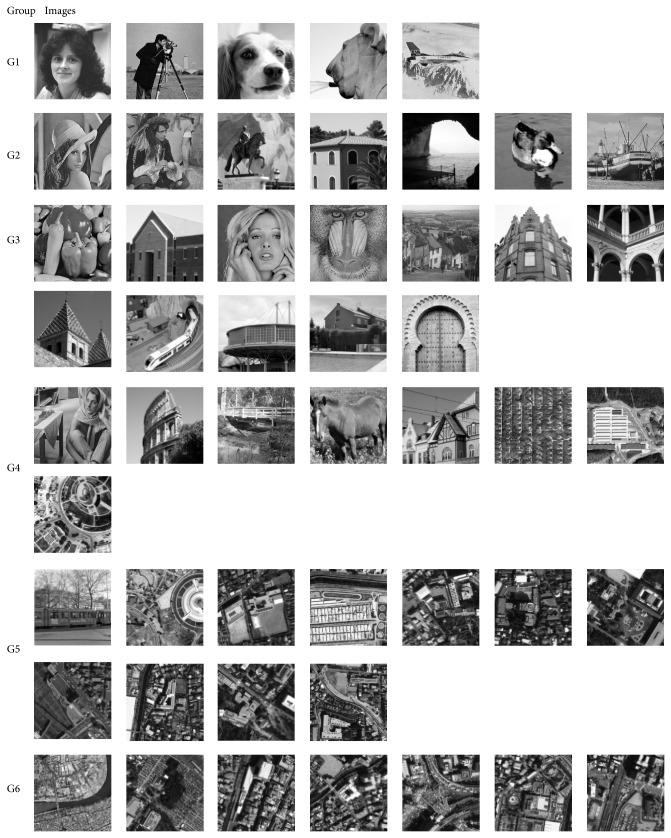
Group of classified images.

The images are classified into one of the six groups (G1, G2,…,G6) according to the* F_*MEAN value and the corresponding classification rule is shown in Table 5. For more clarity, the classified images are categorized in Figure 9 according to their groups. Finally, a library is build offline by evolving wavelets for each group separately using RCGA with PSNR as the fitness function.

## 4. Experimental Results and Discussion

Initially the images are classified according to the edges and textures using the algorithm discussed in the Section 3.2. The initial classification step provides six groups of images with different texture details. The idea is to evolve wavelet filter coefficients for each individual group both for near-edge and far-edge pixels in an image. Based on the output of the edge detection algorithm, a binary mask is created for the considered image and the binary mask separates the near-edge and far-edge pixels. The next step is to evolve wavelet filter coefficient for the near-edge pixels followed by far-edge pixels. The experiment is repeated for all the images which fall in the same group and the corresponding evolved filter coefficient is stored in the library. The experiment continues with the next group and concludes after evolving filter coefficients for all the six groups. Fifty GA runs are considered for both convolution scheme and lifting scheme and each GA run would consider one of the test images shown in the Figure 9. The GA configuration followed for conducting the experiment is given in Table 3.

Thus we have created an optimal wavelet library suitable for image compression for each class of images. For compressing an arbitrary image, its optimal wavelet filter coefficients need to be selected from the prestored library based on its* F*_MEAN value which serves as an index for the selection of wavelets. We have evolved 9 filter coefficients for convolution scheme as the 2-variable evolver failed in most situations to produce a better wavelet than the CDF 9/7. For lifting scheme single variable is evolved as the 5-variable evolver failed because of its NIL constraint situation. The evolved wavelet libraries for both global and local filter are shown in Table 6.

**Figure 10:**
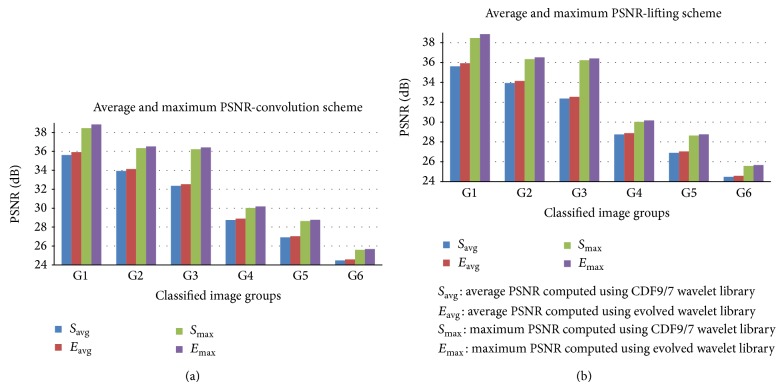
Comparison of quality metrics of the reconstructed image using CDF 9/7 and evolved filter coefficients; (a) average and maximum PSNR for convolution scheme; (b) average and maximum PSNR for lifting scheme.

**Figure 11:**
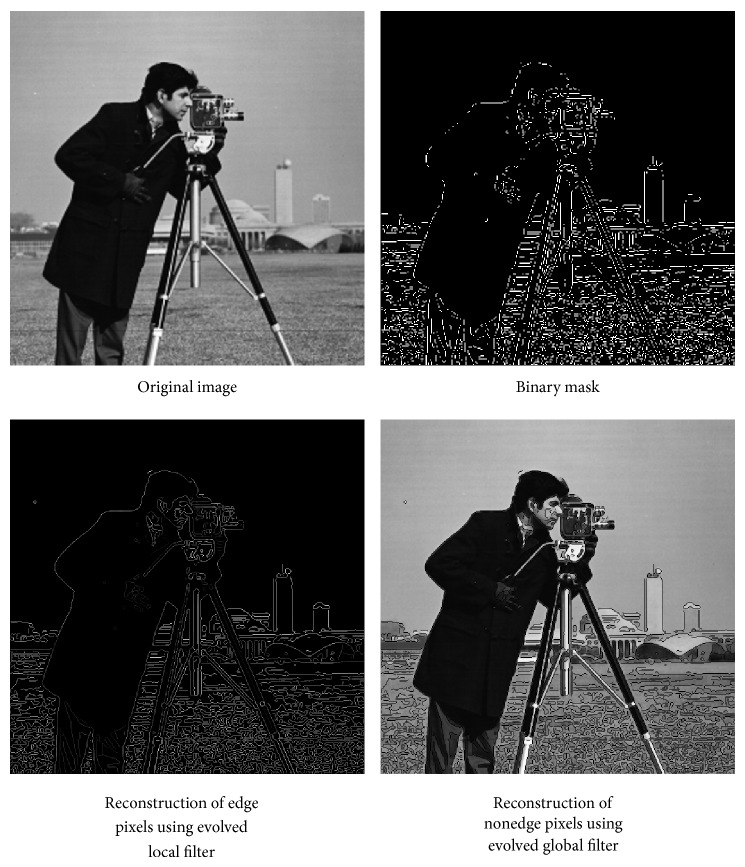
Reconstruction of images using global and local filters.

**Figure 12:**
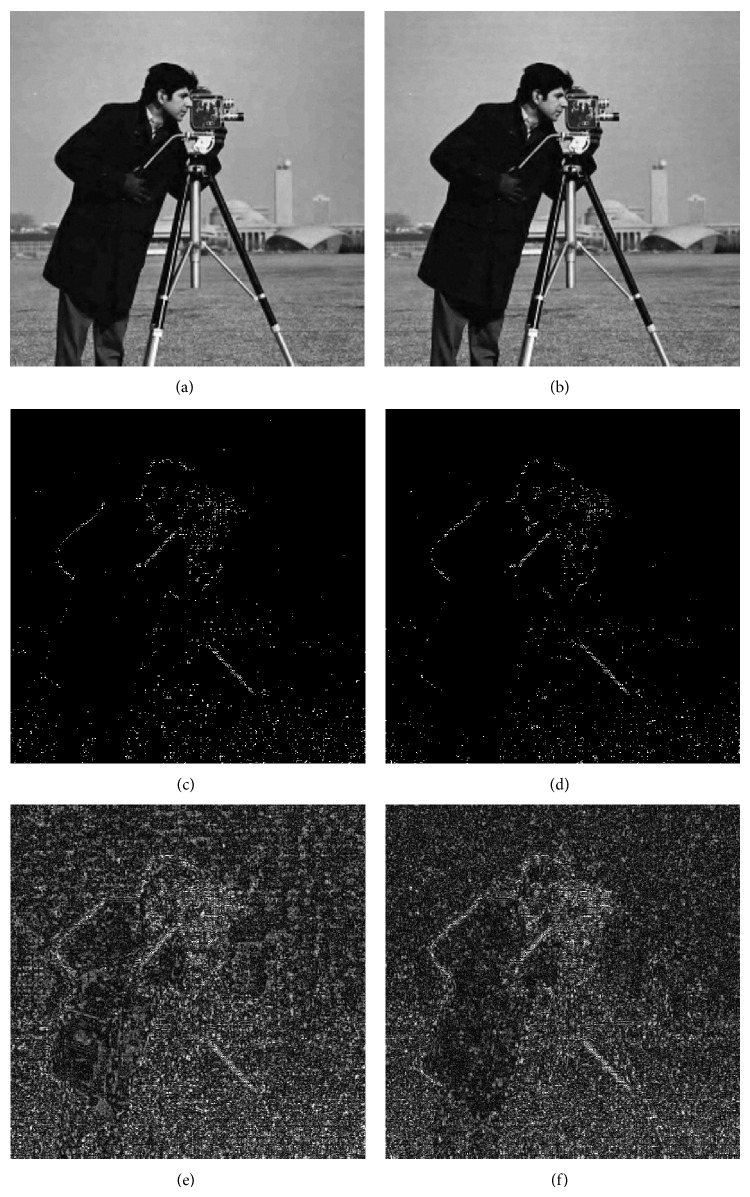
Comparison of evolved and CDF 9/7 filters. ((a), (b)) Reconstructed image using evolved and CDF 9/7 filter coefficients. ((c), (d)) Difference image using evolved and CDF 9/7 filter coefficients. ((e), (f)) Enhanced difference images for better visibility.

The comparison of the quality measures in convolution and lifting schemes are shown in Figure 10.  Figure 11 shows the images reconstructed using global and local evolved filters and Figure 12 shows the comparison between images reconstructed using CDF 9/7 and evolved filters coefficients.

The ES based wavelet optimization algorithm discussed by Salvador et al. [7, 8] focused on hardware implementation choosing FPGA as the base fabric. The existing ES is modified to suit the hardware implementation with a hardware efficient mutation operator and it was tested for both floating point and fixed point arithmetic. Our focus is to improve the quality of reconstruction (PSNR) by evolving wavelet filter coefficients for image subgroups based on the texture and edges. Also, the same evolved filter coefficients may not suit for all image groups and hence we evolve different filters even if the improvement is marginal for the first level of decomposition. The improvement is best pronounced as the decomposition level increases.  Table 7 compares the optimization methodology and improvement in the results in terms of PSNR.

## 5. Conclusion and Future Work

Thus a lossy image compression method with improved performance compared to the CDF 9/7 based compression has been designed. The experimental results of Hybrid subband decomposer for the selected test images show significant improvement in the average PSNR and the maximum PSNR value for the reconstructed image subjected to quantization. Thus evolving wavelets for each group of images classified according to the* F*_MEAN value is robust for performing lossy image compression. The evolved wavelets show an average improvement of 0.31 dB and a maximum improvement of 0.39 dB under convolution scheme. Under lifting scheme the evolved wavelets show an average improvement of 0.27 dB and a maximum improvement of 0.35 dB. Apart from using PSNR as the quality metric, the wavelets can be evolved by also considering SSIM and EC for the fitness measure to further improve the performance of compression. As extrinsic evolution of filter coefficients takes large amount of time, intrinsic evolution can be carried out by implementing an optimized light weight GA core on an FPGA platform so that filter coefficients can be evolved in lesser amount of time and hence make it suitable for adaptive systems.

## Figures and Tables

**Table 1:** CDF 9/7 filter coefficients.

*n*	Analysis filter coefficients		Synthesis filter coefficients	
	Low	High	Lowr	Highr
1	0.02674875741080976	0.09127176311424948	0.09127176311424948	0.02674875741080976
2	−0.01686411844287495	−0.05754352622849957	−0.05754352622849957	0.01686411844287495
3	−0.07822326652898785	−0.5912717631142470	0.5912717631142470	−0.07822326652898785
4	0.2668641184428723	1.115087052456994	1.115087052456994	−0.2668641184428723
5	0.6029490182363579	−0.5912717631142470	0.5912717631142470	0.6029490182363579
6	0.2668641184428723	−0.05754352622849957	−0.05754352622849957	−0.2668641184428723
7	−0.07822326652898785	0.09127176311424948	−0.09127176311424948	−0.07822326652898785
8	−0.01686411844287495			0.01686411844287495
9	0.02674875741080976			0.02674875741080976

**Table 2:** Optimum solutions obtained by the proposed GA.

Test function	Minimum value	Theoretical solution	Optimum solutions obtained by the proposed GA
Rosenbrock function *f*(x, y) = 100(y−x^2^)^2^ + (1 − x^2^), −2 < (*x*, *y*) < 2	0.024433341	(1, 1) (0.896, 0.803)	*x* = 0.9959105 *y* = 1.0074636

De Jong's function in 2D *f*(x, y) = x^2^ + y^2^, −5 < (*x*, *y*) < 5	0.847917595	(0, 0)	*x* = 0.0863404 *y* = 0.0189192

Rastrigin's function *f*(x, y) = 10 × 2 + [x^2^ − 10cos⁡(2πx)] + [y^2^ − 10cos⁡(2πy)], −5 < (*x*, *y*) < 5	0.423719237	(0, 0)	*x* = 0.00539928 *y* = 0.04605763

**Table 3:** Initial configuration of four different GAs.

Parameter variables	Convolution filter		Lifting scheme	
	9	2	5	1
Wavelet DWT implementation	Matlab wavelet toolbox	Matlab wavelet toolbox	Matlab wavelet toolbox	Matlab wavelet toolbox

Fitness function	PSNR	PSNR	PSNR	PSNR

Generations	500	500	500	500

Permitted filter coefficient values	[21.0,1.0]	[−0.2,0.4] [−0.1,0.3]	[−1.5,1.5]	[1.0,1.3]

Population initialization	CDF9/7	CDF9/7	CDF9/7	CDF9/7

Population size	200	200	200	200

Selection operator	Random/stochastic uniform	Random/stochastic uniform	Random/stochastic uniform	Random/stochastic uniform

Elite	10	10	10	10

Crossover rate	0.5/0.8	0.5/0.8	0.5/0.8	0.5/0.8

Recombination	Wright's heuristic	Wright's heuristic	Wright's heuristic	Wright's heuristic

Mutation operator	Gaussian	Gaussian	Gaussian	Gaussian

Mutation standard deviation	0.3	0.3	0.3	0.3

Mutation shrink rate	1.0	1.0	1.0	1.0

**Table 4:** *F*_MEAN values for 50 test images.

Image	*F*_MEAN
P1	10.5147
P2	5.5117
P3	16.1949
P4	18.9663
P5	17.2253
P6	3.9881
P7	15.0666
P8	15.5465
P9	22.0394
P10	17.5639
P11	20.7364
P12	14.3209
P13	16.4834
P14	26.1350
P15	18.7598
P16	11.6503
P17	34.2640
P18	18.4811
P19	29.4208
P20	25.5023
P21	15.2356
P22	6.8231
P23	16.8230
P24	14.8521
P25	7.5128
P26	10.8964
P27	11.5614
P28	13.0462
P29	16.7697
P30	8.3708
S1	28.6052
S2	25.3179
S3	30.5507
S4	68.5516
S5	32.3629
S6	30.7848
S7	50.1749
S8	27.4554
S9	48.5618
S10	45.0343
S11	51.6971
S12	44.3086
S13	47.0612
S14	32.6130
S15	34.4422
S16	38.5398
S17	42.2794
S18	46.8646
S19	42.5832
S20	44.8287

**Table 5:** Classification rule.

Complexity level	*F*_MEAN
G1	0–9.999
G2	10–14.999
G3	15–19.999
G4	20–29.999
G5	30–44.999
G6	Above 45

**Table 6:** Evolved wavelet libraries.

		Complexity level					
		G1	G2	G3	G4	G5	G6
9	Global	0.054629	0.012605	0.032392	0.029695	0.036221	0.03662
		−0.02452	−0.00053	−0.02269	−0.01927	−0.02057	−0.0227
		−0.06516	−0.07454	−0.07889	−0.07895	−0.07514	−0.08703
		0.334343	0.262207	0.279217	0.280296	0.309677	0.295225
		0.632333	0.69317	0.714242	0.707114	0.627418	0.683349
		0.143108	0.109115	0.032392	0.096019	0.147287	0.090116
		−0.09349	−0.04232	0.089464	−0.05766	−0.05681	−0.0556
		−0.54933	−0.56726	−0.05808	−0.5389	−0.58923	−0.53448
		0.99999	0.98762	−0.53136	0.99761	0.999996	0.997501
	Local	−0.14134	−0.11646	−0.09367	−0.09367	−0.16327	−0.09198
		−0.08997	−0.06448	−0.06013	−0.06013	−0.0764	−0.05909
		0.550634	0.555133	0.537672	0.537672	0.58579	0.53853
		0.97280	0.99986	0.93826	0.92341	0.999997	0.96312
		0.550634	0.555133	0.537672	0.537672	0.58579	0.53853
		−0.06832	−0.08022	−0.07966	−0.07966	−0.08858	−0.08863
		−0.32981	−0.2571	−0.28254	−0.28254	−0.31979	−0.30657
		0.626812	0.702748	0.703591	0.703591	0.621365	0.670795
		−0.32981	−0.2571	−0.28254	−0.28254	−0.31979	−0.30657

1	Global	1.293053	1.286464	1.263407	1.259675	1.273145	1.265296
	Local	1.290432	1.291972	1.276267	1.256783	1.27437	1.265312

**Table 7:** Comparison of other implementation methodologies.

Method	EA	Implementation	Seed	Image details	Max. PSNR value (dB)
Salvador et al. [7]	Simpler ES	Hardware: FPGA	Random Gaussian	Fingerprint image	36.9672
Salvador et al. [8]	Simpler ES	Hardware: FPGA	Random and CDF 9/7	FVC2000 fingerprint image	37.0146
Our Approach	Modified GA	Extrinsic (software)	CDF 9/7 with random noise	Versatile image set	38.8502
CDF 9/7	—	Extrinsic (software)	—	Versatile image set	38.4563

**Table 8:** Comparison of PSNR value for the 50 test images: convolution scheme.

G1		G2		G3		G4		G5		G6	
S_PSNR	E_PSNR	S_PSNR	E_PSNR	S_PSNR	E_PSNR	S_PSNR	E_PSNR	S_PSNR	E_PSNR	S_PSNR	E_PSNR
38.45631	38.85018	36.33535	36.5171	34.98924	35.13623	27.72834	27.85837	27.02089	27.14499	21.94497	22.04289
36.70699	36.92435	34.0207	34.27553	32.11272	32.29091	28.82493	28.96298	27.4896	27.62331	24.94645	25.08053
34.09777	34.40723	33.87194	34.10702	32.24662	32.28161	30.0196	30.16959	27.4311	27.58621	24.53254	24.62632
34.34908	34.71995	32.03045	32.2167	36.22086	36.40928	28.33613	28.46977	28.64238	28.76313	25.58698	25.6771
34.4365	34.71073	33.30052	33.44693	34.40422	34.5957	27.3513	27.52124	25.68813	25.78846	24.31887	24.39961
35.60933	35.92249	32.6565	32.89177	31.85983	32.00643	29.62113	29.76113	27.8452	27.96407	24.99085	25.13824
		35.29927	35.48987	30.58591	30.74712	29.22614	29.37626	27.91306	28.0401	25.05632	25.13908
		33.93067	34.13499	29.93214	30.11399	28.86373	28.97372	27.04521	27.16217	24.48243	24.58625
				31.5267	31.7053	28.74641	28.88663	25.98266	26.12673		
				30.20841	30.34334			25.63449	25.74427		
				31.11664	31.37826			25.32481	25.4685		
				33.14686	33.36982			26.91068	27.03745		
				32.36251	32.5315						

**Table 9:** Comparisons of quality measures of standard and evolved wavelet: lifting scheme.

G1		G2		G3		G4		G5		G6	
S_PSNR	E_PSNR	S_PSNR	E_PSNR	S_PSNR	E_PSNR	S_PSNR	E_PSNR	S_PSNR	E_PSNR	S_PSNR	E_PSNR
38.93267	39.20322	35.68218	35.86366	34.04916	34.22233	27.55357	27.67565	26.85292	26.95729	21.85391	21.97152
37.14987	38.04428	33.31568	33.5484	31.45538	31.67873	28.596	28.76565	27.2943	27.40592	24.85093	24.95663
36.32518	36.53486	33.46184	33.75286	31.86329	31.99898	29.85774	29.98002	27.28039	27.4149	24.42999	24.51269
35.16321	35.43679	31.56559	31.86296	34.82506	34.95671	28.03903	28.19076	28.38014	28.52757	25.4236	25.65941
36.02053	36.12321	32.62601	32.91051	33.64955	33.79002	27.18739	27.36666	25.54373	25.64735	24.21356	24.31126
36.71829	37.06847	32.18305	32.32529	31.35368	31.49754	29.20575	29.32815	27.63069	27.74617	24.90763	24.99951
		34.29313	34.5091	30.18976	30.38405	28.87345	29.01003	27.62497	27.72541	24.93023	25.02014
		33.30393	33.53897	29.56117	29.60132	28.52454	28.66757	26.8556	26.98592	24.37284	24.49017
				31.25065	31.39991	28.47968	28.62306	25.83868	25.97445		
				29.91406	30.1079			25.5617	25.69367		
				30.86037	30.99509			25.16427	25.30552		
				32.48319	32.62449			26.72976	26.85311		
				31.78794	31.93809						

## Appendix

### PSNR Values for Images

The 50 test images are grouped under six groups (G1, G2, …, G6) and they are compressed and reconstructed using standard and evolved wavelet transform in the convolution scheme and their PSNR values are compared in Table 8. For lifting scheme the corresponding comparison is depicted in Table 9.
